# Parental Ethnic Identity and Its Influence on Children’s Oral Health in American Indian Families

**DOI:** 10.3390/ijerph18084130

**Published:** 2021-04-14

**Authors:** Anne R. Wilson, Rachel L. Johnson, Judith Albino, Luohua Jiang, Sarah J. Schmiege, Angela G. Brega

**Affiliations:** 1 Department of Pediatric Dentistry, School of Dental Medicine, University of Colorado Anschutz Medical Campus, Aurora, CO 80045, USA; 2 Department of Biostatistics and Informatics, Colorado School of Public Health, University of Colorado Anschutz Medical Campus, Aurora, CO 80045, USA; rachel.johnson@cuanschutz.edu (R.L.J.); sarah.schmiege@cuanschutz.edu (S.J.S.); 3 Centers for American Indian and Alaska Native Health, Colorado School of Public Health, University of Colorado Anschutz Medical Campus, Aurora, CO 80045, USA; judith.albino@cuanschutz.edu (J.A.); ANGELA.BREGA@CUANSCHUTZ.EDU (A.G.B.); 4 Department of Epidemiology, School of Medicine, University of California Irvine, Irvine, CA 92697, USA; lhjiang@hs.uci.edu

**Keywords:** social identification, oral health, American Indians, dental caries, health behavior, pediatric

## Abstract

Objectives: To examine the relationship between ethnic identity and oral health knowledge, beliefs, behavior, and outcomes in American Indian families. Methods: Secondary data were analyzed for 579 parent–child dyads in a randomized controlled trial aimed at reducing early childhood caries in a Northern Plains tribal community. Data included demographic characteristics; parental ethnic identity; oral health knowledge, beliefs, and behavior; and parental/pediatric oral health outcomes. Ethnic identity was assessed using two measures: perceived importance of tribal identity and tribal language proficiency. We examined the association of baseline ethnic identity with baseline and longitudinal oral health measures. Results: At baseline, importance of tribal identity was significantly associated with several oral health beliefs, and one’s locus of control measure (external-chance). Baseline scores on importance of tribal identity were also associated with one’s oral heath belief (perceived severity), the same locus of control measure, and oral health knowledge and behavior over the three years of study follow up. Tribal language proficiency was not associated with any study measures at baseline, although it was associated with parental oral health status over the three years. Conclusions: Ethnic identity was associated with a range of oral health constructs expected to influence American Indian children’s oral health.

## 1. Introduction

In the United States, American Indian and Alaska Native groups are disproportionately affected by persistent oral health disparities. In particular, young Native children under six years of age experience disproportionate rates of dental decay, commonly referred to as early childhood caries [1,2,3,4,5,6]. Early childhood caries is nearly three times higher for Native children than for non-Hispanic white children [2].

These findings highlight the need for greater understanding of social determinants specific to Native populations as an underpinning of oral health promotion initiatives. The literature suggests that ethnic identity may be associated with health-related outcomes among Native groups. Ethnic identity has been described as the subjective identification with and sense of belonging to one’s ethnic group, or for Native people, one’s tribe of origin [7]. Indicators of ethnic identity include use of the language of one’s ethnic group, perceived importance of ethnic group membership in relation to one’s self concept, identification with one’s cultural values, and participation in one’s cultural practices [8]. For Native groups, the salutogenic value of ethnic identity merits further investigation as part of health promotion efforts for oral health disparities [9,10,11].

For Native populations burdened by extreme health disparities, ethnic identity has been explored relative to general health and the related influence on preventive behaviors and behavioral compliance with medical recommendations [9,12,13,14]. Some studies have shown that a strong sense of ethnic identification is associated with higher rates of optimal health beliefs and behaviors [15,16,17,18,19,20] and better health outcomes [9,20,21,22,23,24]. In other studies, an inverse relationship between ethnic identity and health-promoting behaviors has been discerned. In particular, strong ethnic identity has been linked to suboptimal health behaviors related to tobacco and drug use [9,14] and reduced adherence with cancer screening recommendations [12,13,25,26,27]. 

The connection between Native ethnic identity and oral health is not well understood and minimally studied in relation to parental influences on children’s health. As is the case for general health, the relationship between ethnic identity with oral health varies and at times may function as a facilitator for oral health promoting behaviors or as a deterrent for the same behaviors. In a previous study, the association of ethnic identity with parental oral health knowledge, beliefs, and behaviors was evaluated as part of a randomized controlled trial aimed at reducing early childhood caries [28]. Findings provided evidence of a cross-sectional association of perceived importance of one’s tribal identity with strong oral health knowledge and positive oral health-related beliefs. These results were consistent with studies in other health areas suggesting a positive association of ethnic identity with oral health-related attitudes [15,16,17,18,19,20,21,22]. However, results showed no relationship between perceived importance of tribal identity with oral health behavior, and a negative relationship between tribal identity with parental and pediatric oral health status. These results highlight the complex relationship between ethnic identity and oral health in Native populations [28]. 

The objective of this secondary analysis was to examine the association of ethnic identity with longitudinal measures of parental oral health knowledge, beliefs, behavior, and with parental and pediatric oral health status based on data from the randomized controlled trial entitled “Promoting Behavior Change for Oral Health in American Indian Mothers and Children” (PBC) [29,30]. This study was conducted on a Northern Plains reservation with a population of approximately 20,000 and the majority being members of a single tribe [31]. Using baseline and longitudinal data from the three-year PBC trial, the primary aim of this study was to evaluate the role of ethnic identification with oral health constructs that may influence parental engagement in their young children’s oral health. Given that prior research has examined only the cross-sectional association of ethnic identity with oral health-related factors in Native people, this work provides crucial insight into the possibility that ethnic identity might influence Native children’s oral health over time. 

## 2. Materials and Methods

### 2.1. Participants and Procedures

The PBC study was conducted with parents of Native children aged 0–3 years who resided on or near the participating reservation in the Northern Plains [29,30]. The tribal region is the second largest in the United States and characterized by extremes in climate, poverty, geographic isolation, and limited access to dental services from a paucity of clinical sites and dental providers [29,30]. For participating adults, the designation of “parent” was used. Five hundred and seventy-nine parent–newborn dyads were enrolled. The aim of the PBC study was to address the high prevalence of early childhood caries [32] using motivational interviewing as an interventional approach for Native parents that was consistent with tribal values of respect and autonomy [29,30]. After three years, the motivational interviewing intervention led to improved parent knowledge but did not lead to a change in parent oral health behaviors or progression of early childhood caries [30].

Study participants were enrolled at four towns within or close to the reservation including the Indian Health Service hospital, project field office, powwows, health fairs, tribal colleges, and local community events on the reservation and in Rapid City, South Dakota. Study enrollment was initiated on July 2011 and completed in March 2014, with the last follow-up assessment in January 2017. Participating children were evaluated at three follow-up time points (12, 24, and 36 months) by calibrated dental examiners masked to group assignment to identify tooth surfaces that were decayed, missing, or filled (dmfs) [29,33]. All participants received respondent compensation for each visit through the study duration [29,30].

Participating parents completed the Basic Research Factors Questionnaire (BRFQ) at the three follow-up assessments [34]. Survey items solicited information about parents’ ethnic identity, oral health knowledge, beliefs, and behavior; parents’ assessment of their own and their children’s oral health status; and sociodemographic characteristics of participating parents and children. Survey data were collected using an audio computer-assisted self-interviewing system touch-screen audio computer-assisted self-interviewing with narration by a tribal project staff member [29,35]. The system allowed participants to replay a survey item, mute the audio, and remove the headphones. The ACASI data collection methods have been shown to improve reliability of reported health-related behaviors among other AI groups [35]. The parent completed the survey at the time of the child’s evaluation which took approximately 45 to 60 min [29].

Approval for these secondary analyses of data from the PBC project was obtained from the participating tribe’s research review board and the Colorado Multiple Institutional Review Board of the University of Colorado (Protocol 17-0287). Parents provided written consent and HIPAA authorization. For parents under age 18 years, consent was obtained from their parents/legal guardians. All study activities were conducted in accordance with accepted ethical standards for human subjects research.

### 2.2. Measures

Analyses used baseline and longitudinal data from the BRFQ to examine the association of ethnic identity with measures of parental oral health knowledge, beliefs, and behavior as well as oral health outcomes. These measures have been validated in Native populations [36,37,38,39].

#### 2.2.1. Ethnic Identity

Two BRFQ items from the baseline time point were used to assess parents’ ethnic identity. These items were adapted from the Special Diabetes Program for Indians Healthy Heart Project [40] and developed in accordance with orthogonal cultural identification theory [41] and the Bicultural Ethnic Identity Scale [10]. The first item assessed the perceived importance of one’s ethnic identity, asking parents to indicate “how important is it to you that you maintain your tribal identity and your tribe’s values and practices?” Response options were: 1 = not at all; 2 = a little; 3 = somewhat, 4 = very much. Because few parents selected the two lowest response options, the categories of “not at all” and “a little” were combined in all analyses. The second item assessed tribal language proficiency, asking parents “how well do you speak your tribal language?” Response options were: 1 = I don’t speak my tribal language; 2 = I speak it a little, but not very well; 3 = I speak it moderately well; 4 = I speak my tribal language very well. In all regression analyses, the categories of “moderately” and “very well” were combined, due to a small number of parents selecting these response options. Because the correlation between the two items was only of moderate strength (r = 0.30), we considered the two variables as separate components of ethnic identity rather than a single factor.

#### 2.2.2. Parental Oral Health Knowledge

Sixteen BRFQ items assessed parents’ knowledge of pediatric oral health and recommended parental oral health behaviors [6,37]. Responses were coded as correct or incorrect, with “don’t know” coded as incorrect. The overall oral health knowledge measure was computed as the percentage of questions answered correctly.

#### 2.2.3. Parental Oral Health Beliefs

Items from the BRFQ measured several constructs from established health behavior theories that highlight the importance of health beliefs on health behavior.

##### Health Belief Model (HBM)

BRFQ items captured five constructs from the extended Health Belief Model (HBM) including perceived severity of dental caries, perceived susceptibility to dental problems, perceived barriers to and benefits of recommended oral health behavior, and self-efficacy (i.e., confidence that one can successfully engage in recommended behaviors) [42,43]. Application of the HBM suggests that parents are more likely to engage in recommended oral health behaviors if they believe their children are susceptible to caries, that caries is a severe condition, that there are few barriers and many benefits to recommended parental oral health practices, and that they are capable of engaging in these behaviors [37,38]. For perceived susceptibility, severity, barriers, and benefits, the BRFQ included 3–5 items each, all of which used a scale of 1–5. The average of items for each construct was computed, with higher numbers reflecting a greater degree of the construct. One item was poorly correlated with the other two and excluded from calculation of the perceived susceptibility score (children can get cavities as soon as their first tooth comes in). Fourteen BRFQ items assessed parents’ self-efficacy. The items used a scale of 1–5, with higher numbers reflecting greater self-efficacy. As most parents selected the highest possible score for many of the items, the overall self-efficacy score was computed as the number of items for which the highest score was selected. The overall self-efficacy score had a range of 0–14.

##### Locus of Control (LOC)

LOC theory suggests that parents who believe they personally have control over their children’s oral health are more likely to engage in behaviors resulting in better outcomes for their children. If parents believe their children’s oral health depends on powerful others, such as the dentist, or mere chance, they are less likely to proactively adhere to recommended behaviors. Nine BRFQ items assessed parents’ beliefs regarding the source of control over their children’s oral health [44] Three items each assessed the three different types of LOC: internal, external-powerful other, and external-chance. For each LOC type, the average of the three items assessing that domain was computed. Scores ranged from 1–5, with larger numbers reflecting greater endorsement of items in that domain.

#### 2.2.4. Parental Oral Health Behavior

Thirteen BRFQ items assessed parental oral health behaviors. For each item, responses were coded as adherent or non-adherent with recommendations for positive oral health practices. The overall behavioral adherence score was computed as the percentage of recommended behaviors with which parents reported being adherent [45].

#### 2.2.5. Oral Health Outcomes

Three indicators of oral health were used in this analysis. Two measures assessed the oral health of participating children, the children’s dmfs scores and their parents’ reported assessment of their children’s oral health status. The third measure assessed the parents’ self-reported oral health status. Pediatric oral health outcomes were not assessed at baseline as teeth were not present in newborns.

Two BRFQ items measured the oral health status of parents and children. The items were adapted from the National Survey of Children’s Health [46] and measured parental assessment of (1) their children’s oral health status and (2) parents’ own self-reported oral health status. Oral health status was rated using a five-point scale: 1 = excellent, 2 = very good, 3 = good, 4 = fair, 5 = poor and analyzed as a continuous variable. 

Using data from oral evaluations, the dmfs score was computed for each child and had a potential range of 0 to 88 tooth surfaces [33]. Tooth surfaces were counted as decayed when there was visible missing tooth structure from disease. Teeth were counted as missing if determined to be prematurely lost due to disease rather than trauma or other causes unrelated to disease. Inspection was visual, without radiographs or probing of teeth [33].

#### 2.2.6. Demographic Characteristics

The BRFQ included items assessing parent and child age, sex, race, and ethnicity. Other items for parents included tribal affiliation, highest grade completed, household income, and employment status. Education was coded using a four-point scale: 1 ≤ high school graduate, 2 = high school graduate or GED, 3 = some college or vocational school, 4 = college degree or more. Income was measured as the total pre-tax household income for the prior year and was coded using a five-point scale that ranged from 1 = ≤ $10,000 to 5 ≥ $40,000. A category for missing income data was included to account for the 23% of participants who declined to provide information about their household income.

### 2.3. Data Analysis

Analyses examined the association of ethnic identity with oral health knowledge, beliefs, behavior, and outcomes at baseline and over time. The alpha level was considered significant if less than 0.05. All variables were summarized using the mean and standard deviation (SD) for continuous variables and frequency and percentage for categorical variables. The two ethnic identity variables were summarized as continuous variables in the descriptive tables and for regression analyses treated as nominal categorical variables with three categories (as described above) for easier interpretation.

Using baseline data, the relationships of the two ethnic identity variables with demographic characteristics were examined using one-way Analysis of Variance. We conducted multiple linear regression models using baseline data to examine the cross-sectional association between each of the ethnic identity variables and the oral health constructs (oral health knowledge, beliefs, behavior, and outcomes). Baseline parental age and income were included as covariates in these models.

To determine whether baseline ethnic identity was associated with oral health over time, we examined the associations of baseline ethnic identity with the oral health constructs at three follow-up time points (12, 24, and 36 months). By analyzing the longitudinal associations this way, we tested the possibility that ethnic identity might influence oral health knowledge, beliefs, behaviors, and outcomes at a later point in time. The oral health constructs were modeled longitudinally using random intercept linear mixed models (LMMs) for all variables except dmfs. Other potential covariance structures accounting for the intraclass correlations among repeated measures of the variables were also tested, including an unstructured covariance, and first order autoregressive covariance. However, based on the Akaike information criterion, none of the covariance structures fit the data better than the random intercept model. Because dmfs is a highly skewed variable with many zeros, a generalized linear mixed model (GLMM) was employed to fit the dmfs outcome with a negative binomial distribution and a random intercept for each participant. The GLMMs with Poisson distribution and first order autoregressive covariance or unstructured covariance structure were also tested but did not fit the model as well as the GLMM described above. For all longitudinal regression models, the primary independent variables were time (categorized at each time point) and baseline perceived importance of tribal identity or baseline reported tribal language proficiency. Covariates included parent’s age and income at baseline, child’s gender, and child’s age in months at each visit. The interactions between the ethnic identity items and time were also tested in these models but none were significant. Therefore, the interactions were removed from the final models. 

## 3. Results

### 3.1. Participant Characteristics

Baseline characteristics of the sample are presented in Table 1. Among the 579 enrolled dyads, 562 parents (97%) responded to survey questions about ethnic identity at baseline. Children were 0.7 months old on average (approximately 3 weeks old) and the mean age for parents was 25.1 years old (range 15 to 49 years). Almost all parents were female (97%) and American Indian (96%), and approximately three-quarters self-reported being members of the participating Northern Plains tribe. A small number of parents (5%) reported being Latino. Forty percent of parents reported that they did not complete high school, with 7% having completed a college or more advanced degree. More than half of the parents reported being unemployed and having an annual household income of less than $10,000. 

### 3.2. Baseline Association of Parent Demographics with Ethnic Identity

To examine the relationship between ethnic identity and parents’ demographic characteristics at baseline, associations between these two sets of variables are summarized in Table 2. Only educational attainment was positively associated with ethnic identity (*p* < 0.001). Participants with lower levels of education reported lower perceived importance of maintaining their tribal identity compared to those with higher levels of education.

### 3.3. Baseline Association of Perceived Importance of Tribal Identity with Oral Health Constructs

After adjusting for parental age and income, three of the five constructs of the extended HBM were significantly related at baseline to the perceived importance of maintaining tribal identity (Table 3). Parents with higher levels of tribal identification perceived pediatric oral health problems to be more severe (*p* = 0.010), perceived fewer barriers to (*p* = 0.023) and more benefits of good oral health behaviors (*p* = 0.014) in comparison with parents with lower levels of tribal identification. Parents with higher levels of tribal identification were less likely to endorse the belief that their children’s oral health was a matter of chance (*p* = 0.005). Although not significant, parents with stronger tribal identity adhered to a moderately larger percentage of recommended parental oral health behaviors (*p* = 0.07). The importance of maintaining tribal identity was not significantly associated with oral health knowledge, perceived susceptibility and self-efficacy, internal LOC, powerful others LOC, or parental oral health status at baseline. Pediatric oral health outcomes were not assessed as teeth were not present in newborns at baseline.

### 3.4. Baseline Association of Tribal Language Proficiency with Oral Health Constructs

As shown in Table 4, at baseline, tribal language proficiency was not a significant predictor of the oral health constructs and not associated with any of the other oral health constructs reflecting parents with a higher level of proficiency in one’s tribal language skills did not differ with those reporting a lower proficiency level.

### 3.5. Longitudinal Association of Perceived Importance of Tribal Identity with Oral Health Constructs

The adjusted mean values of each construct at each time point, with reference to the importance of maintaining tribal identity, revealed significant associations for several of the oral health measures. Parents reporting higher levels of tribal identification at baseline perceived pediatric oral health problems to be more severe than did parents who reported lower levels of tribal identification over the three years of study follow up (*p* = 0.048). Additionally, parents with higher baseline levels of tribal identity were less likely to endorse the belief that their children’s oral health is a matter of chance over time than were parents with lower levels of tribal identification (*p* = 0.017). Finally, parents perceiving higher importance of tribal identity at baseline had higher levels of oral health knowledge (*p* = 0.033) and behavioral adherence (*p* = 0.009) over the three years of study follow up than did parents for whom tribal identity was not as important. Baseline importance of tribal identity was not significantly associated with the other HBM and LOC measures, self-reported oral health status, or dmfs over the three years of study follow up.

### 3.6. Longitudinal Association of Tribal Language Proficiency with Oral Health Constructs

The adjusted mean values of each construct at each time point, with reference to tribal language proficiency, demonstrated a significant association with parental oral health status over the three-year follow-up period (*p* = 0.003). Parents who were able to speak their tribal language moderately or very well reported better oral health status than did those who reported less skill with their tribal language. Tribal language proficiency was not associated with any other measures of parental oral health knowledge, beliefs, behavior, or pediatric outcomes including dmfs.

## 4. Discussion

Prior to the 1770s, American Indian and Alaska Native people maintained cultural practices and values intrinsic to their well-being. After this time, Native groups in the United States experienced forced colonization and relocation, treaties requiring assimilation, removal of children from families and placement in boarding schools to instill Euro-American values, language, and practices, and loss of tribal land. In 1975, after centuries of subjugation, the Indian Self-Determination and Education Assistance Act gave tribes authority over their welfare and management of federal funds [11]. The 1975 Act has contributed to tribal management of education and health-care systems and development of culture and language revitalization efforts. Nonetheless, after centuries of sociopolitical oppression extreme socioeconomic, educational, environmental and health disparities endure [11]. Given these challenges, the role of ethnic identity in health promotion through mobilization of social and cultural resources as health-enhancing factors has become an emerging area of research.

The degree to which parental tribal identity contributes to health promotion efforts in addressing oral health disparities among Native children has not been fully established. Using cross-sectional and longitudinal data from a large-scale randomized control trial, ethnic identity among American Indian parents was assessed in relation to oral health knowledge, beliefs, behaviors, and outcomes. To our knowledge, this study is one of only a few to examine these relationships and the first to examine the longitudinal association of ethnic identity with oral health knowledge, beliefs, behaviors, and oral health outcomes over time.

Longitudinal findings from this study reflect that parental ethnic identity was related to a range of oral health constructs over time. Consistent with expectations, over the study duration, parents with higher levels of tribal identification demonstrated higher levels of oral health knowledge and behavioral engagement compared to parents with lower levels of tribal identity. Parents perceived their children’s oral health to be related to their individual efforts and not a matter of chance, and perceived dental caries is a severe health concern. Despite these positive associations, parents did not perceive fewer barriers and more benefits of good oral health behaviors compared to parents with lower levels of tribal identification. Study outcomes inconsistent with the expected theoretical direction of behavioral models may reflect the deleterious effects of prevailing social determinants in reservation environments. In particular, barriers to accessing oral health care in tribal communities with longstanding extreme poverty contributes to a higher sense of fatalism and unfavorable oral health behaviors and outcomes [47,48]. Given the extreme socioeconomic, educational, environmental, and health disparities on this and other isolated reservation communities, intractable social determinants may predominate over cultural influences associated with health-promoting oral health behaviors. 

With respect to tribal language proficiency, results from longitudinal assessments did not demonstrate associations between parental tribal language proficiency for nearly all oral health constructs encompassing knowledge, beliefs, behavior, and pediatric oral health outcomes. The only contributory finding was an association between parents reporting higher ability to speak their tribal language also reporting better oral health status. Findings corroborate the complex relationship between one’s native language with oral health outcomes. 

In American Indian and Alaska Native groups, facility with one’s Native language has been assessed relative to knowledge and behavioral compliance with preventive recommendations for general health concerns. The literature, however, is unclear regarding the role of facility with one’s Native language and health behaviors. Use of one’s Native language at home has been associated with reduced compliance with recommended health screenings for colorectal cancer among reservation-dwelling Native populations [13]. Conversely, in a Hopi community from a Southwestern reservation, findings failed to support the concept that primary use of one’s tribal language was a barrier to preventive health screening [49,50]. Investigators concluded that tribal language use may not function as a barrier to preventive health knowledge and behaviors due to tribal bilingualism, as English is acquired simultaneously with Hopi during childhood. As such, the advantage of being bilingual may be additive rather than subtractive in health-promoting behavior [49,50]. In the Northern Plains, biculturalism was associated with positive health behaviors associated with physical activity [17]. The advantage of biculturalism over monoculturalism involving both minority and majority cultures and American Indian populations has been attributed to generating a higher sense of self-efficacy [17].

In this longitudinal study conducted in a Northern Plains tribal community, ethnic identity was associated with a range of oral health constructs expected to influence Native children’s oral health at baseline and in longitudinal assessments. Despite these findings, ethnic identity is a multifaceted determinant and was evaluated using two items; one of these, tribal language proficiency, proved not to be a significant factor. While some baseline results from this study were corroborated in previous studies on the Navajo Nation reservation, others were not [28]. These results highlight the complex relationship between ethnic identity and oral health in Native populations and that ethnic identity may differ at the community level. As an inherent limitation of a localized analysis, factors unique to the Northern Plains tribal community may not be generalizable to other Native groups. Subsequent studies are merited that include a range of Native communities. Future studies are recommended that provide a broader exploration of ethnic identity including language biculturalism and collective resilience as factors found to be related to shared identity and cultural practices in tribal groups [11]. Much remains to be learned about the relationship between ethnic identity and the salutogenic contribution to health promotion efforts as part of research and clinical interventions addressing longstanding oral health disparities in Native communities.

## 5. Conclusions

Although assumptions have been made regarding associations between ethnic identity and health-related influences in Native people, evidence has not been available regarding oral health and parental influences on children’s outcomes. Findings from this longitudinal study in a large cohort provide initial support for the potential causal association between ethnic identity and parental oral health knowledge, beliefs, behavior, and outcomes in a Northern Plains tribe.

## Figures and Tables

**Table 1:** Baseline dyad characteristics for parents completing the ethnic identity items (N = 562).

Parent Characteristics	Mean (SD) or N (%)
Age (months)	25.1 (5.4)
Gender: Female	546 (97.2%)
Race and ethnicity	
American Indian	540 (96.1%)
Hispanic or Latino	30 (5.3%)
Member of Oglala Sioux Tribe	419 (74.6%)
Highest grade completed	
<High school graduate	224 (39.9%)
High school grad or GED	140 (24.9%)
Some college or vocational	159 (28.3%)
College degree or more	39 (6.9%)
Income	
<$10K	291 (51.8%)
$10–<$20K	57 (10.1%)
$20–<$30K	46 (8.2%)
$30–<$40K	16 (2.8%)
≥$40K	22 (3.9%)
Income missing	130 (23.1%)
Employment status	
Full- or part-time employment	100 (17.8%)
Full- or part-time student	47 (8.4%)
Homemaker	67 (11.9%)
Unemployed	285 (50.7%)
Other (retired, disabled, medical leave)	38 (6.8%)
Relationship to child	
Mother	539 (95.9%)
Father	14 (2.5%)
Other	1 (0.2%)
**Child Characteristics**	
Age (months)	0.7 (0.9)
Gender: Female	288 (51.2%)
Race and Ethnicity	
American Indian	562 (100.0%)
Hispanic or Latino	50 (8.9%)

**Table 2:** Baseline associations between parent demographic characteristics and ethnic identity.

	Importance of Tribal Identity			Tribal Language Proficiency		
	N	Mean (SD)	*p*-Value	N	Mean (SD)	*p*-Value
Age quartiles (years)			0.13			0.12
15–20	110	3.5 (0.8)		120	1.8 (0.7)	
21–23	114	3.5 (0.8)		119	1.6 (0.7)	
24–28	153	3.4 (0.9)		155	1.5 (0.6)	
29+	137	3.6 (0.7)		136	1.7 (0.7)	
Highest grade completed			<0.001			0.13
<High school graduate	211	3.3 (0.9)		222	1.6 (0.7)	
High school grad/GED	128	3.5 (0.9)		135	1.6 (0.6)	
Some college/vocational	158	3.7 (0.7)		158	1.6 (0.6)	
College degree or more	38	3.6 (0.8)		36	1.9 (0.9)	
Income			0.38			0.57
Income missing	115	3.4 (0.8)		126	1.6 (0.7)	
<$10K	284	3.4 (0.9)		287	1.6 (0.7)	
$10K to <$20K	55	3.6 (0.8)		56	1.7 (0.6)	
$20K to <$30K	44	3.5 (0.8)		45	1.6 (0.6)	
$30K to <$40K	15	3.9 (0.3)		15	1.7 (0.8)	
≥$40K	22	3.6 (0.8)		22	1.7 (0.8)	

**Table 3:** Baseline associations between importance of tribal identity and parental oral health constructs.

	Not at All/a Little (N = 81)	Somewhat (N = 99)	Very Much (N = 355)	Adjusted *p*-Value *
Oral health knowledge	74.2 (12.0)	74.9 (12.6)	77.2 (12.2)	0.492
Extended Health Belief Model				
Perceived susceptibility	3.1 (1.1)	2.9 (1.0)	2.8 (1.0)	0.138
Perceived severity	4.2 (0.8)	4.3 (0.8)	4.5 (0.7)	0.010
Perceived barriers	2.3 (0.9)	2.1 (0.9)	2.0 (0.8)	0.023
Perceived benefits	4.1 (0.9)	4.3 (0.8)	4.4 (0.6)	0.014
Self-efficacy	7.4 (4.0)	7.6 (3.5)	8.4 (3.5)	0.053
Locus of control				
Internal	4.0 (1.0)	4.1 (1.0)	4.2 (0.8)	0.106
External: powerful others	2.4 (1.2)	2.3 (1.1)	2.1 (1.1)	0.265
External: chance	2.7 (1.2)	2.5 (1.1)	2.2 (1.0)	0.005
Behavioral adherence	57.3 (19.4)	57.9 (17.6)	61.8 (18.6)	0.070
Parental oral health status **	3.1 (1.2)	3.4 (1.0)	3.4 (1.1)	0.139

* Analyses were adjusted for parent age and income; ** baseline outcome data were only available for parents and pediatric outcome measures are not reported.

**Table 4:** Baseline associations between tribal language proficiency and parental oral health constructs.

	Not at All (N = 249)	A Little (N = 261)	Moderately or Very Well (N = 41)	Adjusted *p*-Value *
Oral health knowledge	76 (12.7)	76.6 (12.4)	71.6 (16.9)	0.108
Extended Health Belief Model				
Perceived susceptibility	3.0 (1.1)	2.8 (1.0)	2.9 (1.1)	0.174
Perceived severity	4.4 (0.7)	4.4 (0.7)	4.2 (1.0)	0.164
Perceived barriers	2.1 (0.9)	2.0 (0.8)	2.1 (0.8)	0.525
Perceived benefits	4.3 (0.8)	4.4 (0.7)	4.3 (0.8)	0.848
Self-efficacy	7.9 (3.5)	8 (3.6)	8.3 (3.6)	0.936
Locus of control				
Internal	4.2 (0.8)	4.2 (0.9)	4.0 (1.1)	0.453
External: powerful others	2.2 (1.1)	2.2 (1.1)	2.4 (1.3)	0.631
External: chance	2.4 (1.2)	2.2 (0.9)	2.6 (1.3)	0.052
Behavioral adherence	58.1 (18.7)	61.4 (18.8)	61.1 (17.3)	0.221
Parental oral health status **	3.5 (1.0)	3.4 (1.2)	3.0 (1.1)	0.106

* Analyses were adjusted for parental age and income; ** baseline outcome data were only available for parents and pediatric outcome measures are not reported.

## Abbreviations

BRFQ	Basic Research Factors Questionnaire
dmfs	Decayed, missing and filled tooth surfaces
GLMM	Generalized linear mixed model
HBM	Health Belief Model
LMMs	Linear mixed models
LOC	Locus of control
PBC	Promoting Behavioral Change for Oral Health in American Indian Mothers and Children
SD	Standard deviation
